# Lung cancer induced in mice by the envelope protein of jaagsiekte sheep retrovirus (JSRV) closely resembles lung cancer in sheep infected with JSRV

**DOI:** 10.1186/1742-4690-3-94

**Published:** 2006-12-19

**Authors:** Sarah K Wootton, Michael J Metzger, Kelly L Hudkins, Charles E Alpers, Denis York, James C DeMartini, A Dusty Miller

**Affiliations:** 1 Division of Human Biology, Fred Hutchinson Cancer Research Center, Seattle, Washington 98109, USA; 2 Department of Pathology, University of Washington, Seattle, Washington 98195, USA; 3 Molecular Diagnostic Services, Westville 3630, South Africa; 4 Department of Microbiology, Immunology, and Pathology, Colorado State University, Fort Collins, Colorado 80523, USA

## Abstract

**Background:**

Jaagsiekte sheep retrovirus (JSRV) causes a lethal lung cancer in sheep and goats. Expression of the JSRV envelope (Env) protein in mouse lung, by using a replication-defective adeno-associated virus type 6 (AAV6) vector, induces tumors resembling those seen in sheep. However, the mouse and sheep tumors have not been carefully compared to determine if Env expression alone in mice can account for the disease features observed in sheep, or whether additional aspects of virus replication in sheep are important, such as oncogene activation following retrovirus integration into the host cell genome.

**Results:**

We have generated mouse monoclonal antibodies (Mab) against JSRV Env and have used these to study mouse and sheep lung tumor histology. These Mab detect Env expression in tumors in sheep infected with JSRV from around the world with high sensitivity and specificity. Mouse and sheep tumors consisted mainly of well-differentiated adenomatous foci with little histological evidence of anaplasia, but at long times after vector exposure some mouse tumors did have a more malignant appearance typical of adenocarcinoma. In addition to epithelial cell tumors, lungs of three of 29 sheep examined contained fibroblastic cell masses that expressed Env and appeared to be separate neoplasms. The Mab also stained nasal adenocarcinoma tissue from one United States sheep, which we show was due to expression of Env from ovine enzootic nasal tumor virus (ENTV), a virus closely related to JSRV. Systemic administration of the AAV6 vector encoding JSRV Env to mice produced numerous hepatocellular tumors, and some hemangiomas and hemangiosarcomas, showing that the Env protein can induce tumors in multiple cell types.

**Conclusion:**

Lung cancers induced by JSRV infection in sheep and by JSRV Env expression in mice have similar histologic features and are primarily characterized by adenomatous proliferation of peripheral lung epithelial cells. Thus it is unnecessary to invoke a role for insertional mutagenesis, gene activation, viral replication, or expression of other viral gene products in sheep lung tumorigenesis, although these processes may play a role in other clinically less important sequelae of JSRV infection such as metastasis observed with variable frequency in sheep.

## Background

JSRV is the cause of a contagious lung cancer in sheep and goats that occurs in many countries worldwide [1]. Disease progression leading to death may take years in adult sheep but lung tumors can appear in as little as 10 days in experimentally-infected animals [2]. Disease and death is primarily the result of tumor growth and the production of excess lung fluid that lead to breathing difficulty [3]. The disease was originally called jaagsiekte, an Afrikaans term derived from "jaag" (to chase or hunt) and "siekte" (sickness), as diseased sheep appear to have been chased even when at rest and particularly when driven. JSRV-associated lung cancer has been called sheep pulmonary adenomatosis, ovine pulmonary carcinoma, or ovine pulmonary adenocarcinoma, the latter being the currently accepted name [3].

Several mechanisms have been proposed for JSRV oncogenesis, including the expression of an oncogene carried by the virus, by insertional activation of host cell oncogenes, or by inactivation of host cell tumor suppressor proteins. The Env protein of JSRV can transform a variety of cultured cell types [4-9] and can induce lung tumors in mice [10] and in sheep [11], indicating that Env is the primary determinant of oncogenesis. Expression of JSRV Env in mouse lung was achieved by nasal administration of a replication-defective AAV6 vector that encodes only the JSRV Env protein. Env-induced tumor number showed a linear correlation with vector dose [12], indicating single-hit kinetics of tumor formation and arguing against a requirement for host oncogene activation by vector insertion into the host cell genome in these mice. Others have attempted to find common integration sites for JSRV in tumor tissue from sheep to identify oncogenes that might be activated by JSRV, but only one common integration site (2 proviruses 2.5 kb apart out of 37 studied) has been identified, no activated oncogene has been found, and tumors appear multiclonal [13,14]. Localization of the gene encoding the receptor for JSRV cell entry, Hyal2, to a tumor suppressor locus in human chromosome 3 (3p21.3) led to speculation that inactivation of Hyal2 by Env might play a role in oncogenesis [4]. However, mouse Hyal2 is not functional as a receptor for JSRV nor does it bind JSRV Env [4,15-17], yet JSRV Env is able to induce tumors in mice [10], indicating that Env interaction with Hyal2 is not required for tumorigenesis. Together these results indicate that JSRV oncogenesis is mediated entirely by Env through pathways independent of Env interaction with the virus receptor Hyal2.

Here we have addressed the question of how closely tumors induced by JSRV Env in mice resemble those induced by JSRV in sheep, in part to determine if the oncogenic activity of Env can entirely account for the disease observed in sheep. To facilitate these studies we have generated high-specificity high-sensitivity mouse Mab against JSRV Env that detect tumor cells expressing Env in sheep with JSRV disease from North and South America, Africa, and Europe. JSRV is not known to be associated with tumors originating in tissues other than the lung in JSRV-infected sheep, but we wanted to see if JSRV Env could induce tumors in other tissues in mice. Tail vein injection of the AAV6 vector encoding JSRV Env resulted in the production of various tumor types, showing that JSRV Env can induce tumors in tissues other than the lung in mice. Overall we conclude that the oncogenic activity of JSRV Env displayed in mice can entirely account for the adenomatous proliferative histological phenotype of the vast majority of lung tumors induced in sheep by JSRV.

## Results

### Generation of JSRV Env Mab

We previously showed that administration of an AAV6 vector encoding JSRV Env to the lungs of immunocompetent C57BL/6 mice results in the production of high-titer neutralizing antibodies that can be used to detect Env in histologic sections of tumors induced by JSRV Env in immunodeficient mice [10]. However, due to the polyclonal nature of the antibodies, it is possible that the antibodies recognize tumor antigens in addition to JSRV Env, and there was low-level background binding of the antibodies to lung tissue from mice not expressing Env.

To make Env-specific antibodies, we generated Mab against the surface (SU) domain of JSRV Env as follows. C57BL/6 mice were exposed to a replication-defective AAV6 vector encoding JSRV Env (ARJenv) [10] by nasal aspiration. Antibody titers in blood were measured every week until they plateaued, at which time one mouse received an injection of a hybrid JSRV Env SU-human IgG constant fragment protein, produced and purified as described [16], followed by a second injection three weeks later. Three days after the last injection, the mouse was killed and spleen cells were used to make monoclonal cell lines by fusion with mouse myeloma cells. Cell clones were screened for production of antibodies against JSRV Env or human IgG by ELISA assay. Clones producing antibodies against human IgG were discarded and 8 clones isolated from different master plates that produced antibodies against JSRV Env were chosen for further analysis. These Mab brightly stained cultured rat cells that expressed JSRV Env (data not shown).

### Mab staining of lung tumors from mice

**Figure 1:**
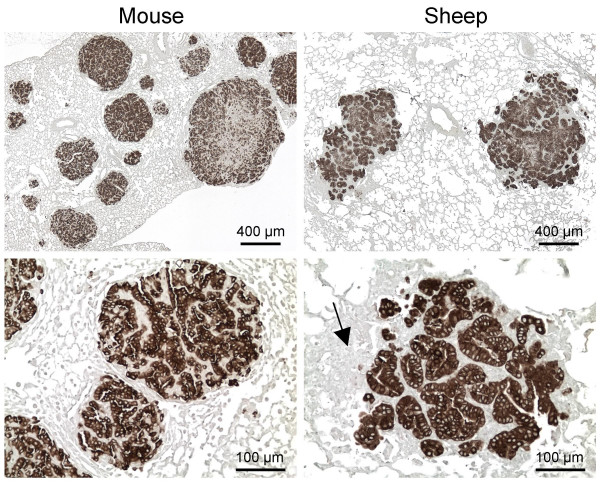
**Mab staining of mouse and sheep lung tumors**. Left panels are from a mouse exposed 2 months previously to an AAV6 vector encoding JSRV Env (ARJenv) [10], and right panels are from sheep 85RS14 (Table 1) that was experimentally infected with JSRV. Sections were stained with the Env Mab C9 and were counterstained with methyl green. Arrow in lower right panel indicates inflammatory  cells that do not stain for Env expression.

All eight of the selected Mab brightly stained tumors in histologic sections of lungs from immunodeficient mice exposed to an AAV6 vector that expresses JSRV Env, ARJenv [10], with little to no staining of histologically-normal lung tissue (Fig. 1, left panels; data not shown). Notably, Env expression appears to be required for tumorigenesis in this system, because we never observed masses of epithelial cells (tumors) that did not stain with the Env Mab in sections of lungs from different animals that in total contained over 500 Env^+ ^tumors. Mab clones B3 and C9 were chosen for subsequent studies. These two Mab appear to recognize different epitopes since optimal antigen recognition in histological sections requires an antigen retrieval step for the C9 Mab but not for the B3 Mab. However, both Mab recognize the same cells in serial sections of JSRV Env-induced lung tumors in mice (not shown). Neither Mab recognized histologically-similar lung tumors induced in mice by urethane [18] (samples kindly provided by Alvin M. Malkinson; data not shown). In addition to their histological similarity, both Env- and urethane-induced tumors are primarily composed of cells that express the alveolar type II cell marker surfactant protein C and do not expresses the non-ciliated bronchiolar Clara cell marker CC-10 [10,18]. These data indicate that the Mab are specific for JSRV Env and do not recognize mouse tumor antigens expressed by this type of tumor.

Ovine enzootic nasal tumor virus (ENTV) is closely related to JSRV, and like JSRV, the Env protein of ENTV can induce lung tumors in mice following AAV6 vector-mediated Env gene transfer [12]. The SU domain of JSRV Env, against which the Mab were made, is 96% identical to that of ENTV, and we tested whether the Mab would recognize ENTV Env also. Indeed, the Mab recognized ENTV Env in mouse tumors induced by administration of an AAV6 vector that expresses only the ENTV Env protein [12].

### Mab staining of tumors from sheep

We next tested the Mab for staining of lung tumors in sheep with confirmed JSRV disease following experimental infection with the JS7 strain of JSRV. Lung tumors in these sheep were brightly stained by the Mab B3, C9, or a mixture of the two, with no staining of histologically-normal lung tissue (Table 1; Fig. 1, right panels). The appearance of many of the Mab-stained sheep lung tumors was remarkably similar to that of mouse lung tumors induced by exposure to ARJenv, the AAV6 vector that only encodes JSRV Env (Fig. 1, left panels). The majority of lung tumors in sheep and mice appeared as adenomas consisting of well-differentiated epithelial cells. There was more lung inflammation in the immunocompetent sheep in comparison to the immunodeficient mice (Fig 1), as might be expected. However, the Mab clearly differentiated  tumor cells from Env-negative immune  cells, connective tissue, and myxomatous tissue [19,20] that were often  found within and around the sheep tumors.

**Table 1:** JSRV Env-antibody staining of histologic sections of lung tissue from sheep

Country of origin	Sheep number	antibody			
		
		B3	C9	B3+C9	polyclonal
USA (experimentally- infected)	84RS17	+	+		
	84RS18			+	+
	85RS1	+			
	85RS14	+	+		
	85RS22			+	+
					
USA (naturally- infected)	84RS28	+	+		
	85RS65			+	+
	98RS1	+	+		
	98RS3			+	+
	99RS27			+	+
	99RS33			+	+
					
Peru	81R15	+	+		+
	81R16	+	+		
	81R22	+	+		+
	81R71	+	+		+
	81R78	+	+		+
					
Spain	B-96/00			+	+
					
Kenya	92K3			+	+
					
South Africa	93141			+	
	95195			+	
	95205			+	
	95211			+	
	95226			+	
	95227			+	
	95229			+	
	95234			+	
	95251			+	
	96238			+	
	96269			+	

**Figure 2:**
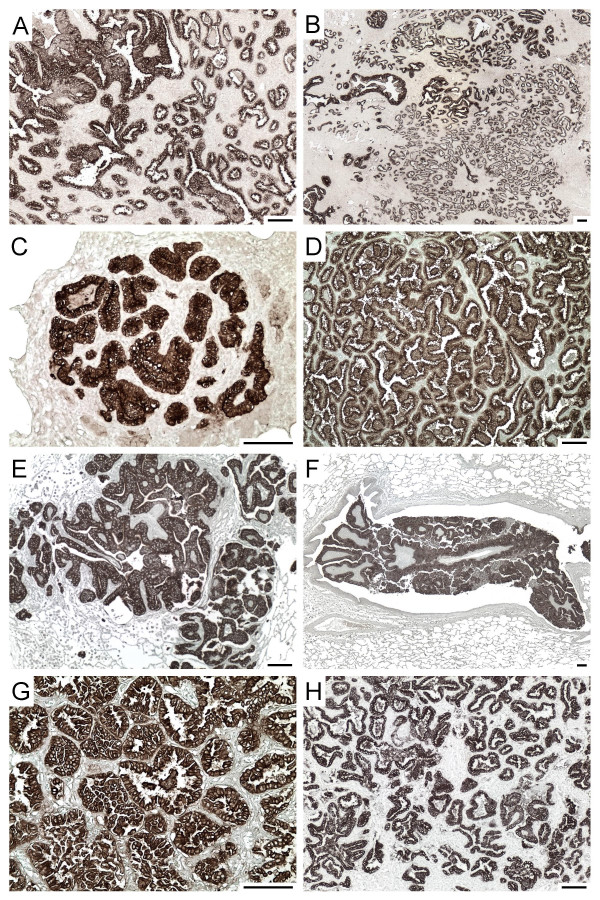
**Mab staining of JSRV-infected sheep lung tumors from around the world**. Sheep numbers and countries of origin are: A, 96238 from South Africa; B, 95234 from South Africa; C, 92K3 from Kenya; D, 81R16 from Peru; E and F, 85RS1 from the USA (experimentally-infected); G, 84RS28 from the USA; and H, B-96/00 from Spain. Sections were stained with Mab B3, C9, or both. Scale bars indicate a distance of 100 μm.

Sequencing of the *env *regions of different JSRV isolates from sheep has revealed several strains that fall into two groups, those from Africa and those from the United Kingdom and United States [21-24]. Our Mab were generated using the JS7 strain of Env [24], an isolate from Scotland, and we wanted to know if the Mab would recognize Env from wild-type strains of JSRV from countries spanning North and South America, Europe and Africa. The Mab recognized tumors in all sheep with JSRV-induced disease from the United States, Peru, Spain, Kenya and South Africa (Table 1, Fig. 2). Because all tumors were recognized by Mab B3, C9, or both, we conclude that the mixture of Mab B3 and C9 is capable of recognizing JSRV Env in tumors caused by wild-type JSRV in multiple geographic regions, in particular, from regions where infection by either of the two major types of JSRV predominate. This may in part result from the fact that the Mab were raised against the SU domain of Env, which is relatively well conserved among JSRV strains that have been sequenced to date.

**Figure 3:**
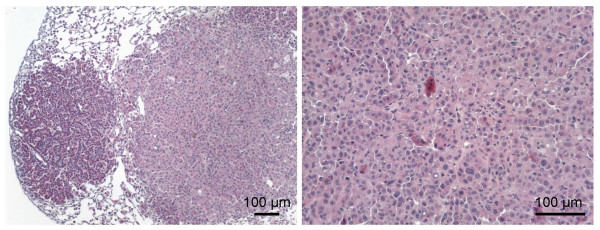
**Adenocarcinoma in a mouse 6 months after exposure to the ARJenv vector**. Tissues were stained with hematoxylin and eosin. Left panel, adenoma (left) and adenocarcinoma (right). Right panel, close-up of an adenocarcinoma showing atypical nuclei.

The majority of sheep tumors examined by Mab staining had the histologic appearance of adenomas with little evidence of anaplasia (Figs. 1 and 2). In contrast, adenocarcinomas were occasionally found in mice at long times (4 to 6 months) after vector administration (Fig. 3). All of these tumors were Env^+ ^as determined by Mab staining (not shown). In some sheep, large adenomatous tumors were present in airways (Fig. 2 panel F), and some mice exhibited similar tumors at long times (4 to 6 months) after exposure to the ARJenv vector encoding JSRV Env (not shown).

**Figure 4:**
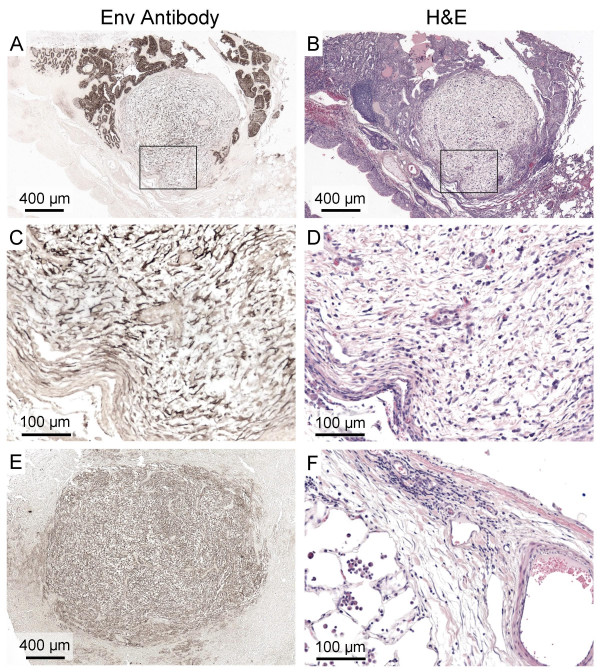
**Fibroblastic cell masses found in some JSRV-infected sheep**. Left panels show Mab C9 staining and right panels show hematoxylin and eosin staining of sheep lung sections. Panels A and B show a round proliferative fibroblastic cell mass (myxomatous tissue) flanked at upper right and left by typical epithelial tumors from South African sheep 96238. Panels C and D show magnified views of the fibroblastic cell mass corresponding to the boxed areas in Panels A and B. Panel E shows Env staining of a large fibroblastic cell mass from naturally-infected United States sheep 85RS65. Panel F shows connective tissue containing fibroblasts at the edge of the lung from South African sheep 93141. These cells were all Env-negative (not shown).

In three sheep (85RS65 and 99RS27 from the United States and 96238 from South Africa) we found proliferative lesions consisting of fibroblasts or other connective tissue cells that expressed Env and that appeared to be separate neoplasms. Low-power views of these lesions revealed relatively round Env^+ ^masses of cells (Fig. 4A, B, E) that were sometimes flanked by typical well-differentiated Env^+ ^epithelial cell tumors (Fig. 4A). High-power views of cells in the fibroblastic areas (Fig. 4C, D) revealed a histological similarity to connective tissue found at the edges of some sheep lungs (Fig. 4F). Such connective tissue lined the lungs and septae projected into the interior of the lungs of some sheep, but none of these tissues stained with Env Mab, including the area shown in Fig. 4F (data not shown). Thus there was a clear differentiation between the streams of Env-negative connective tissue in the lung and the Env^+ ^masses consisting of disorganized immature connective tissue cells. We did not observe such Env^+ ^fibroblastic masses of connective tissue cells in mice transduced with the ARJenv AAV6 vector that encodes JSRV Env, but did observe streams of Env-negative connective tissue by histologic analysis in some mice.

The JSRV Env Mab did not recognize any cross-reacting antigens in lung samples from sheep and goats diagnosed with a variety of diseases that were not the result of JSRV infection. These included lung samples from a sheep and a goat with mucinous goblet cell adenocarcinoma, a sheep infected with ovine lentivirus, and two sheep with inflammatory diseases, one classified as follicular bronchiolitis and the other as lymphoid follicular hyperplasia due to verminous pneumonia.

**Figure 5:**
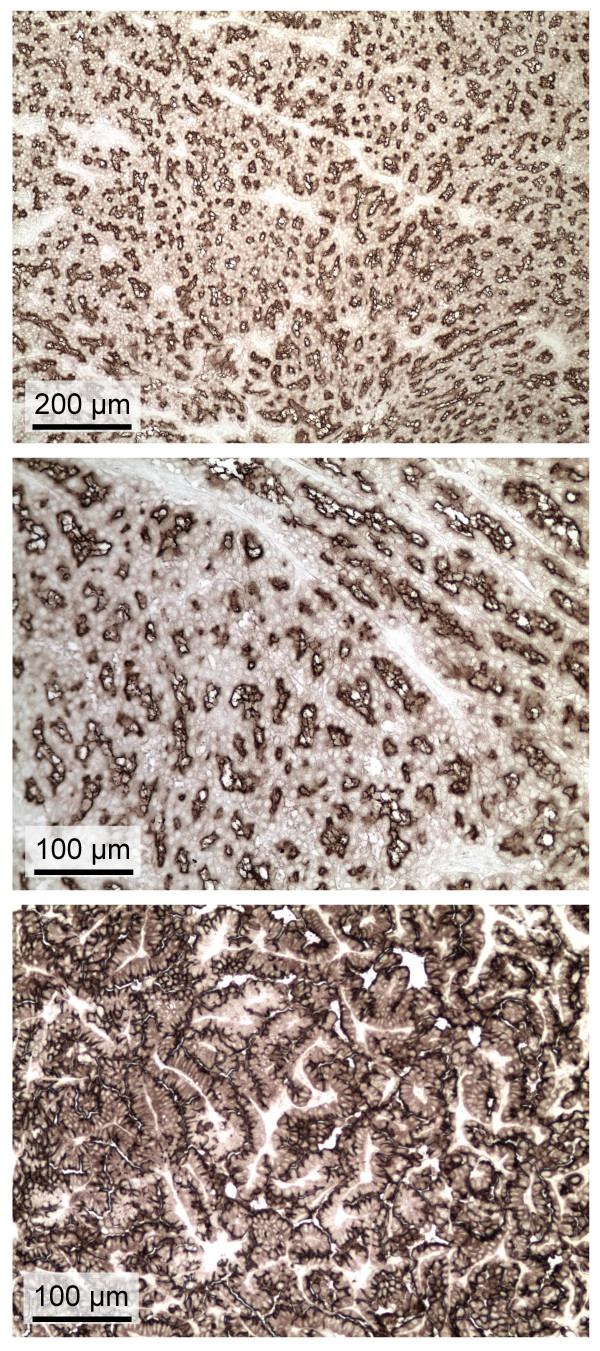
**Mab staining of ENTV Env**. Top and middle panels show Env Mab staining of nasal adenocarcinoma from sheep 99RS39 infected with ENTV. Bottom panel shows Env Mab staining of lung tumor from mouse 5-3 exposed 4 months earlier to an AAV6 vector encoding ovine ENTV Env [12].

Interestingly, the Mab did stain tumor cells in nasal adenocarcinoma from a sheep (no. 99RS39 from the United States) (Fig. 5, top and middle panels), presumably caused by infection with ENTV as it is in Europe. Env staining in the nasal tumor appears to be almost exclusively localized to the apical cell membrane, as opposed to JSRV Env staining which also appears at high levels in the cytoplasm (compare Fig. 5 top and middle panels to Fig. 1, right panels, and Fig. 2). Furthermore, tumors induced in the lungs of mice by an AAV6 vector encoding ENTV Env [12] showed the same apical localization of ENTV Env (Fig. 5, bottom panel) compared to the apical and cytoplasmic localization of JSRV Env (Fig. 1, left panels). PCR amplification followed by direct sequencing of nasal tumor DNA using ENTV-specific primers [25] that amplify a portion of the cytoplasmic tail of Env that is relatively divergent between ovine ENTV (ENTV-1), caprine ENTV (ENTV-2), JSRV, and sheep endogenous retrovirus sequences, revealed that this sheep was indeed infected by a virus with a unique sequence [GenBank: EF184579] most closely related to ENTV-1, with up to 97% identity to existing ENTV-1 sequences. The sequence also contains a 2 bp frameshift in the C-terminus of the Env coding region, a characteristic of the ENTV-1 lineage. These results confirm the suspected presence of ENTV in the United States [26-28].

### Tumors induced in mice following intravenous injection of an AAV6 vector encoding JSRV Env

JSRV DNA and RNA can be detected in lymph nodes, spleen, thymus, bone marrow, and blood cells of sheep infected with JSRV [29,30], and in natural settings systemic infection can be present over long periods without induction of lung tumors [31]. Although oncogenesis originating in tissues other than the lung has not been reported in JSRV-infected sheep, we wanted to determine whether JSRV Env could induce tumors in other tissues.

**Figure 6:**
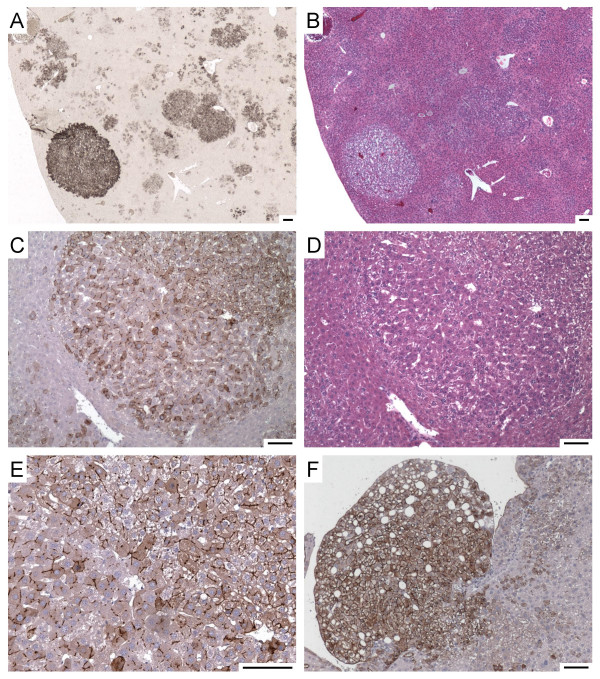
**Hepatocellular tumors induced by intravenous injection of an AAV6 vector expressing JSRV Env**. Panels A and B show low-magnification views of the same area of a liver stained with a mixture of the B3 and C9 Env Mab (light methyl green counterstain) (Panel A) or hematoxylin and eosin (Panel B). Panels C and D show a mixed tumor with adenomatous features in the upper right portion and adenocarcinomatous features in the lower left portion. Panel C shows staining with the Mab (hematoxylin counterstain) and Panel D shows staining with hematoxylin and eosin. Note the compression of liver tissue near the lower left side of the tumor. Panel E shows a high-magnification view of the same tumor in the panel above, with the division between adenoma and adenocarcinoma running from the top left to the bottom right of the panel. Note the linear staining between cells that likely represents Env in bile canaliculi. Panel F shows a tumor with a foamy appearance stained with the Env Mab (hematoxylin counterstain). Scale bars = 100 μm.

**Figure 7:**
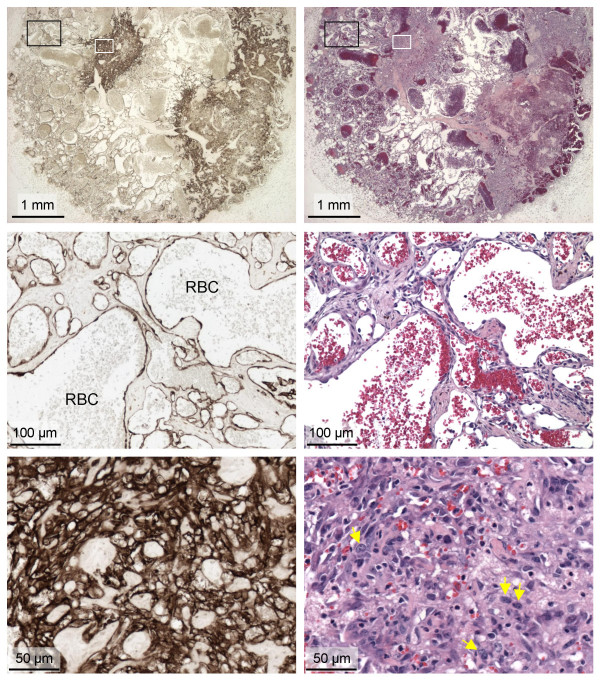
**Hemangiomas and hemangiosarcomas induced by intravenous injection of the AAV6 vector expressing JSRV Env**. Left panels show tumors stained with a mixture of the B3 and C9 Mab (light methyl green counterstain) and right panels show hematoxylin and eosin staining of the same areas shown in the left panels. Top panels show a large cavernous hemangiosarcoma arising in subcutaneous fat. Boxes indicate the areas shown in the middle panels (black boxes) and lower panels (white boxes). Middle panels show an area typical of hemangioma composed of cavernous blood vessels containing residual red blood cells (RBC) that are lined with a single layer of well differentiated, flattened endothelial cells. Note the intense Env Mab staining of endothelial cells but that the collagen and other cells between the vascular spaces do not stain with the Env Mab. Bottom panels show a high-magnification view of an area of hemangiosarcoma comprised of Env^+ ^pleomorphic endothelial cells forming a solid cellular mass. Note examples of pleomorphic nuclei with prominent nucleoli (yellow arrows, lower right panel).

We determined that tail vein administration of an AAV6 vector (ARAP4), that expresses human placental alkaline phosphatase (AP) from the same strong Rous sarcoma virus promoter present in the ARJenv vector [10], led to transduction of multiple tissues in mice, including liver, spleen, heart, kidney, and lung (data not shown). We next administered the ARJenv AAV6 vector to two 1.5-month-old mice. Both mice showed a lack of weight gain starting at 4.5 months of age, showed visible signs of disease starting at 6.5 months of age, and were killed for analysis at 7.5 months of age, 6 months after vector exposure. Only a few of the tissues that can be transduced by an AAV6 vector showed evidence of hyperplasia and/or overt tumor formation. The vector did induce multiple tumors in the liver (Fig. 6A, B). Immunohistochemical staining for Env revealed that Env expression corresponded to the areas of hyperplastic growth, indicating that Env was responsible for lesion formation (Fig. 6A, B). Liver lesions included foci of hepatocellular hyperplasia, adenoma, and rare adenocarcinoma (Fig. 6). In particular, one lesion had a mixed phenotype consisting of adenoma (top right) and adenocarcinoma (bottom left) (Fig. 6C, D, E). A high-magnification view of this tumor showed striking linear staining that likely represents accumulation of Env protein in bile canaliculi (Fig. 6E). Hemangiomas and hemangiosarcomas were observed in multiple fat tissues, most notably in subcutaneous (Fig. 7) and peritesticular (not shown) fat. These lesions ranged from hemangioma (Fig. 7, middle row panels) to hemangiosarcoma (Fig. 7, bottom panels), and all stained positive for JSRV Env expression (Fig. 7 and data not shown). These data show that Env can induce tumors in various cell types besides lung epithelial cells, and some of these tumors have a relatively aggressive histologic appearance.

## Discussion

We have developed Mab against the Env protein of JSRV that give intense staining of lung tumors in sheep infected with JSRV and in mice exposed to an AAV6 vector encoding JSRV Env. These Mab recognized tumors in all JSRV-infected sheep examined (n = 29) from multiple countries. The antibodies did not recognize similar urethane-induced lung tumors in mice. Both urethane- and JSRV Env-induced lung tumors have the same histologic appearance, express the type II alveolar cell marker surfactant protein C and most do not express the Clara cell marker CC-10 [10,18,32]. The Mab did not recognize alveolar type II cell hyperplasia or other cell types in a variety of diseases in sheep that were not the result of JSRV infection. We also found that at least two of the Mab recognized the Env from ovine ENTV in tumors induced in mice by exposure to an AAV6 vector encoding ENTV Env [12], and in a sheep with nasal adenocarcinoma associated with ENTV infection. Together these results indicate that the Mab are highly specific for ovine betaretrovirus Env expression, and would provide a useful diagnostic test for JSRV, and possibly for ENTV as well.

The current accepted nomenclature for lung cancer resulting from JSRV infection is ovine pulmonary adenocarcinoma. The primary reason for its characterization as a malignant disease is because of the observation of metastases consisting of lung tumor epithelial cells, which occurs to a variable extent in sheep [3]. However, the main tumor type we see in JSRV-infected sheep and in JSRV Env-expressing mice is adenoma, consistent with the previous description of the disease as an adenomatosis. Given our results in sheep and in mice, and the fact that what kills these animals is breathing difficulty, it seems the primary effect of JSRV infection, mediated through the Env protein, is to cause proliferation of lung epithelial cells. In JSRV-infected sheep, such proliferation typically increases lung fluid production and thereby facilitates aerosol transmission of the virus produced by epithelial cells. Metastasis may occur as the result of additional genetic changes resulting from virus replication and integration, or resulting directly from Env expression and stimulation of cell proliferation, but these appear not to be the primary effects of virus infection or Env expression.

It is remarkable how little Env Mab staining we observe outside of tumors in mice and sheep. Others have reported similar results in sheep by using polyclonal antibodies to detect JSRV Env or capsid proteins [31-33], but our use here of highly specific Mab that give intense staining of Env-expressing cells helps to rule out the presence of low levels of Env expression outside of tumors. In mice we know that an AAV6 vector encoding AP (ARAP4) can transduce all epithelial cell populations in the airway with relatively high efficiency [34], yet we see no Env staining in large or small airways or in histologically-normal alveoli in mice exposed to the AAV6 vector ARJenv, which like ARAP4 contains a strong Rous sarcoma virus promoter to drive gene expression. It is known that oncoproteins can have both growth-promoting and toxic effects in cultured cells [35], and perhaps only lung stem cells that are the progenitors of tumors can tolerate expression of the potent Env oncoprotein, while Env expression is toxic to the more differentiated cells.

Lack of Env expression outside of tumors in lungs of sheep infected with JSRV is particularly surprising given the presence of replicating virus in the sheep. Perhaps spread of JSRV is inhibited in sheep by an immune response, despite the finding that sheep mount a poor response against JSRV because of immune tolerance induced by proteins made by related endogenous retroviruses [36]. Alternatively, like other simple retroviruses, JSRV may only infect dividing cells, and most potential target cells in the lung are not actively dividing. Most intriguingly, it may be that Env is toxic to most differentiated lung cell types in sheep, as proposed above for mice.

Our results provide further support for the conclusion that the JSRV cell-entry receptor Hyal2 plays no role in sheep tumorigenesis beyond its role as a receptor for virus entry. Mouse Hyal2 does not serve as a cell-entry receptor for retrovirus vectors bearing the JSRV Env protein [4,15-17] nor does it bind the SU domain of JSRV Env [16], yet we have shown here that lung tumors induced in mice by Env expression alone are quite similar to lung tumors induced by JSRV in sheep having a functional Hyal2 virus receptor.

Our results also argue against a role for insertional oncogene activation or insertional mutagenesis in sheep tumorigenesis. An AAV6 vector was used to transfer and express JSRV Env in the mice analyzed here, and tumor induction followed single-hit kinetics [12], a result that is inconsistent with a requirement for insertional events in addition to Env expression for tumorigenesis. In addition, inclusion of an excess of a non-oncogenic AAV6 vector during transduction by the JSRV Env-expressing AAV6 vector reduced the number of tumors [12], again indicating that additional genetic changes that might be caused by the AAV6 vector are not important for tumorigenesis. Together with results shown here that tumors induced by JSRV Env in mice are quite similar to tumors induced by JSRV in sheep, these results indicate that JSRV tumorigenesis is primarily dependent on the oncogenic activity of the JSRV Env protein and does not require genetic changes resulting from JSRV integration.

The main tumor type induced by systemic administration of the AAV6 vector encoding JSRV Env was hepatocellular adenoma. Generation of this non-malignant proliferative tumor is consistent with the activity of JSRV Env in the lung to generate adenomas arising from lung epithelial cells. Given the low frequency of hepatocellular adenocarcinomas following JSRV Env vector administration, it is likely that additional events are required for adenocarcinoma formation, as they appear to be following expression of other oncoproteins such as Myc [37].

Systemic administration of the AAV6 vector encoding JSRV Env to mice induced multiple hemangiomas and some hemangiosarcomas, tumors that arise from uncontrolled and disorganized proliferation of endothelial cells. Endothelial cells in these tumors were uniformly and uniquely stained by the Env Mab, indicating a direct effect of Env on endothelial cells in these tumors.

Oncogenes from other viruses can also induce hemangiomas and have helped elucidate a common pathway for hemangiogenesis that involves phosphatidylinositol 3-kinase (PI3K) activation, downstream activation of Akt, and increased vascular endothelial growth factor production; the latter being a key stimulus for hemangiogenesis. For example, avian sarcoma virus 16 induces hemangiomas and was found to express a viral oncogene derived from the gene encoding the catalytic subunit of PI3K [38]. Viral vectors expressing the viral or cellular forms of the PI3K catalytic subunit could induce hemangiosarcomas in chickens and could transform chicken embryo fibroblasts in culture [38]. Transformation in culture was accompanied by Akt activation and VEGF production, and overexpression of a myristylated form of Akt or VEGF itself could induce hemangiosarcoma formation in chicken embryos [39]. Interestingly, JSRV Env has been shown to transform cultured fibroblasts from mice, rats, and chickens [4-6], and transformation is accompanied by activation of PI3K and Akt in these cells [8,40,41], suggesting that JSRV Env may induce hemangioma formation by activation of the PI3K-Akt-VEGF pathway in mouse endothelial cells.

Another retrovirus that induces hemangiomas is avian hemangioma virus, and like JSRV, this appears to be due to expression of the viral Env protein [42,43]. However, the avian hemangioma virus Env protein shows no similarity to that of JSRV, so it is difficult to predict if the mechanisms of hemangiogenesis are similar. It will be interesting to see if AHV also activates members of the PI3K-Akt-VEGF pathway.

In 10% of JSRV-infected sheep studied we observed masses of Env^+ ^fibroblastic cells that appear to be separate neoplasms. The ability of JSRV Env to transform fibroblasts from several species in tissue culture [4-6], and the uniform Env^+ ^staining of the fibroblastic cell masses in sheep, make it tempting to speculate that these masses represent a novel tumor type. However, the frequent observation of non-neoplastic fibroblast or mesenchymal cell proliferation in response to a number of tissue insults complicates this interpretation. Others have observed similar proliferation of connective tissue in association with epithelial tumors in JSRV-infected sheep [3], but immunohistochemical analysis for Env expression was not performed. We did not see such Env^+ ^fibroblastic masses in mice, but this could simply be due to a lower frequency of the parental cell type in mouse lung. Regardless, these fibroblastic masses were an infrequent occurrence in sheep and thus do not account for the typical disease observed in JSRV-infected sheep.

Nasal administration of the AAV6-Jenv vector to normal C57BL/6 mice results in strong immune responses against Env that limit tumor formation, therefore we have used immunodeficient C57BL/6 Rag-2 mice to model tumor formation by JSRV Env. The question arises whether an immunodeficient mouse is an appropriate model for a disease that occurs in immunocompetent sheep. In fact, expression of multiple endogenous retroviruses related to JSRV in sheep results in immunotolerance toward JSRV infection [36], thus immunodeficient mice appear to be a good model in which to study this intriguing viral disease.

## Conclusion

We have generated Mab against the SU domain of JSRV Env and have shown that these Mab allow robust detection of Env protein synthesis from wild-type strains of JSRV from around the world. The histologic appearance of the majority of lung tumors in sheep infected with JSRV and in mice expressing only the JSRV Env protein is remarkably similar, indicating that Env expression alone can explain much of the disease phenotype in sheep. Indeed, some tumors in mice exhibit a more aggressive adenocarcinomatous histology than do tumors observed in sheep. While JSRV infection in sheep is not known to induce tumors originating in organs other than the lung, systemic expression of JSRV Env in mice induced hepatocellular tumors, hemangiomas, and hemangiosarcomas, showing that Env can induce tumors in cells other than lung epithelial cells. Our results indicate that Env interaction with the virus-entry receptor Hyal2, insertional activation of cellular oncogenes, and insertional mutagenesis do not play major roles in sheep tumorigenesis by JSRV. Overall, the primary effect of JSRV infection is to drive localized proliferation of lung epithelial cells.

## Methods

### Animal studies and safety precautions

Experiments involving mice were performed using procedures approved by the Institutional Animal Care and Use Committee of the Fred Hutchinson Cancer Research Center. Special safety precautions employed during production and use of the AAV6 vectors encoding oncogenic Env proteins were as previously described [10]. All sheep tissue samples were obtained from archival materials collected as part of previously approved studies.

### Mouse immunization protocol for production of Mab

5 × 10^10 ^vector genomes of a replication-defective AAV6 vector expressing JSRV Env (ARJenv) [10] was administered intranasally to lightly anesthetized eight-week-old C57BL/6 mice. Blood samples were collected weekly and sera were screened for the presence of antibodies to JSRV Env protein by ELISA. At 6 weeks post-infection, mice were boosted intraperitoneally with 50 μg JSU-IgG protein in incomplete Freund's adjuvant. JSU-IgG is a hybrid protein consisting of the JSRV Env surface domain (SU) fused to a human IgG Fc [16]. At 9 weeks post-infection, mice were subjected to a second and final boost consisting of 50 μg JSU-IgG (without adjuvant) delivered both intraperitoneally and intravenously.

### Hybridoma generation and characterization of Mab by ELISA

Three days after the last injection, mice were killed and their spleens were removed. Splenocytes were harvested and fused with FOX-NY myeloma cells [44], and hybridomas were selected in medium containing adenine, aminopterin and thymidine as described [44]. Hybridoma supernatants were screened for antibodies against JSRV Env by antigen-dependent ELISA. Briefly, purified JSU-IgG or human IgG was passively adsorbed onto 96 well U-bottom non-tissue culture treated plates (Falcon) at a concentration of 1 μg/ml in Dulbecco's PBS overnight at 4°C. Plates were rinsed with PBS containing 0.05% Tween-20 (PBST) and blocked with PBS containing 5% nonfat milk extract and 2% goat serum for 1 h at 37°C. Antibodies (tissue culture supernatants) were reacted with antigens for 1 h at 37°C, rinsed with PBST, and incubated for an additional hour with a 1:10,000 dilution of HRP conjugated goat anti-mouse IgG (γ chain) (Southern Biotech). Plates were washed and developed using ABST peroxidase substrate (KPL). At 10 and 30 min, the absorbance at 492 nm was determined using a microplate reader. Selected hybridomas were cloned by limiting dilution, and the isotypes of their antibody products were determined by an indirect-capture ELISA. Of the 564 clones that were generated using this vaccination protocol, 52 clones demonstrated specificity of varying degree for JSU-IgG as determined by ELISA. Eight hybridomas that produced antibodies against JSRV Env were selected for further characterization. Of those, 6 were IgG1 (including clones B3 and C9), one was IgG2a and one was IgG2b isotype.

### Immunohistochemistry

Sheep tissues were fixed in 10% formalin, and mouse lung tissue was fixed in 2% paraformaldehyde in phosphate-buffered saline. After fixation tissues were embedded in paraffin wax using an automatic tissue processor and tissue sections (5 μm) were cut and placed on positively charged slides. Samples were deparaffinized and antigen retrieval was performed in a pressure cooker (heat to 120°C, hold for 3 min, allow to cool to 90°C, hold for 3 min) using Antigen Unmasking Solution (Vector Laboratories, Burlingame CA USA). After cooling, endogenous peroxidase was quenched with 3% hydrogen peroxide for 5 min. Mouse IgG was blocked with unconjugated anti-mouse IgG (Vector Laboratories AI-2000) at 1:50 dilution for 15 min. Slides were washed two times for 10 min each with PBS, and medium exposed to hybridoma cells that produce Mab (1:50 dilution in PBS) was incubated with the tissue for 1 h at room temperature. Slides were washed and biotinylated horse-anti-mouse IgG (Vector Laboratories) at a 1:300 dilution was added for 30 min at room temperature. Slides were washed again and incubated with avidin:biotinylated enzyme complex (Vectastain Elite ABC kit; Vector Laboratories). 3,3'-diaminobenzidine tetrahydrochloride (DAB) with nickel chloride enhancement was used as a peroxidase substrate and the sections were counterstained with methyl green.

### Systemic administration of AAV6 vectors

5 × 10^10 ^vector genomes of ARJenv or ARAP4 [45] was administered intravenously to C57BL6/RAG2 mice by tail vein injection. Vectors were suspended in Dulbecco's PBS and administered in a total volume of 0.4 ml. A heating pad was placed in the mouse cage 10 minutes prior to injection in order to dilate tail veins and facilitate delivery of virus. At one-week post infection, mice were given a second injection of 5 × 10^10 ^vector genomes of ARJenv intraperitoneally. Mice were killed 6 months post infection and a full body necropsy was performed. All tissues, with the exception of the lung, were fixed in 2% paraformaldehyde for 48 h, dehydrated, embedded in paraffin, sectioned and stained with hematoxylin and eosin by standard methods. Mouse lungs were perfused with 2% paraformaldehyde and fixed for 4 h. Immunohistochemical staining for JSRV Env was performed as described above. Alkaline phosphatase staining of tissues was performed as described previously [10].

## Competing interests

The author(s) declare that they have no competing interests.

## Authors' contributions

SW generated the Mab, the AAV6 vectors encoding JSRV and ENTV Env, and the AAV6 vector-transduced mice; JD and DY provided sheep samples and helped with data interpretation; MM identified the ENTV virus in the nasal adenocarcinoma sample; KH and CA performed the histologic and antibody staining of mouse and sheep tissues; and AM coordinated the experiments, analyzed the data, and wrote the manuscript. All authors read and approved the final manuscript.
